# Silicone Oil Tamponade–Retina Contact in Highly Myopic Eyes With and Without Encircling Bands: A Computational Fluid Dynamics Study

**DOI:** 10.1167/tvst.11.6.1

**Published:** 2022-06-01

**Authors:** Tommaso Rossi, Giorgio Querzoli, Maria Grazia Badas, Federico Angius, Guido Ripandelli

**Affiliations:** 1 IRCCS Ospedale Policlinico San Martino, Genoa, Italy; 2 DICAAR, Università degli Studi di Cagliari, Cagliari, Italy; 3 IRCCS Fondazione G.B. Bietti per lo Studio e la Ricerca in Oftalmologia ONLUS, Rome, Italy

**Keywords:** Pars plana vitrectomy, retinal detachment, high myopia, posterior staphyloma, encircling band, computational model, computational fluid dynamics, silicone oil tamponade, shear stress, silicone oil

## Abstract

**Purpose:**

To investigate the behavior of silicone oil (SiO) at a steady equilibrium and during saccades in pseudophakic highly myopic eyes with posterior staphyloma with and without an encircling band and compare it to behavior in emmetropic eyes. The SiO–retina contact area and shear stress were calculated by computational fluid dynamics.

**Methods:**

A numerical model of an emmetropic eye and a myopic eye with and without scleral band underwent a saccade of 50°/0.137 s. The vitreous chamber surface was divided into superior and inferior 180° sectors: lens, pre-equator, post-equator, and macula. SiO–retina contact was evaluated as a function of fill percentages between 80% and 90% for standing, 45° upward tilt, and supine patients. Maximum and average shear stress were calculated.

**Results:**

Overall, SiO–retina contact ranged between 40% and 83%; fill percentage varied between 80% and 95%. Neither the encircling scleral band nor the staphyloma significantly affected the SiO–retina contact area, although the presence of a scleral band proved disadvantageous when gazing 45° upward. The inferior retina–SiO contact remained below 40% despite 95% SiO fill. The SS significantly increased at the scleral band indentation and decreased elsewhere. The staphyloma greatly reduced shear stress at the macula.

**Conclusions:**

The presence of a myopic staphyloma reduces shear stress at the macula but does not alter SiO–retina contact significantly. The apposition of a 360° scleral band may reduce SiO–retina contact at least in some postures and increases the SS at the indentation.

**Translational Relevance:**

Assessing SiO–retina contact when vitreous chamber geometry changes according to pathologic or iatrogenic modifications allows accurate prediction of real-life tamponade behavior and helps explain surgical outcomes.

## Introduction

Pars plana vitrectomy involves removal of the vitreous gel and substituting it with gases or liquids, collectively defined “tamponades,” that are intended to contact the broadest possible extension of the retinal surface, especially for retinal detachment repair.

Although all vitreous substitutes exert surface tension at the retinal interface, it is most likely the displacement of aqueous from retinal tears that prevents retinal re-detachment, whereas several other factors play important roles: surface tension, gravity, buoyancy, patient positioning, and vitreous chamber shape.^1^

Previous studies^2^ that have investigated the static and dynamic contact of tamponades have provided important information gained through the use of phantom eyes and the computational fluid dynamics (CFD) of idealized eye models, but far less information has been obtained regarding the behavior of silicone oil (SiO) tamponade in eyes with geometric variations. Variants^3^ of the eye morphology, such as the presence of a posterior staphyloma in pathologic myopia or iatrogenic modifications induced by an encircling scleral band, may significantly alter the vitreous chamber contour from theoretical models, challenging the validity of acquired results.

The present study used CFD to evaluate static and dynamic contact of SiO tamponade with the retinal surface of myopic eyes, in the presence of posterior staphyloma and an encircling band, and compared it to emmetropic geometry.

## Materials and Methods

### CFD Model

**Figure 1.**
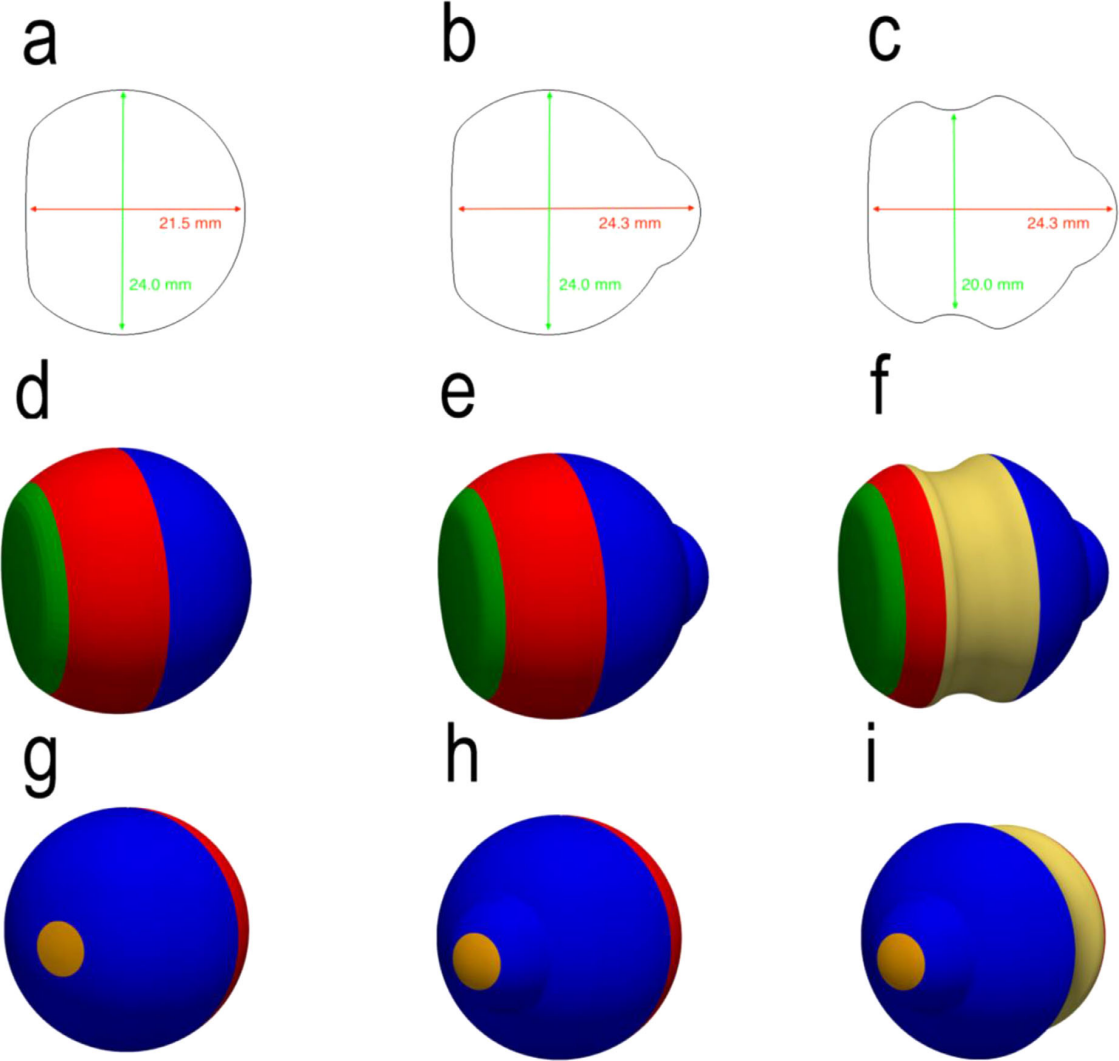
The morphologies investigated: (a, d, g) emmetropic; (b, e, h) myopic staphyloma; and (c, f, i) myopic staphyloma and an encircling scleral band. The *first row* shows sagittal sections, and the *second* and *third rows* show the retinal surface partition used in the present analysis, indicated with different colors: *green*, pars plana; *red*, pre-equatorial retina; *blue*, post-equatorial retina; *pale brown*, macula; *yellow*, cerclage.

The numerical simulations were performed using the open-source library OpenFOAM, which has been successfully adopted in various contexts, including the fluid dynamics of the eye.^3^^–^^6^ Figure 1 shows the three vitreous chamber morphologies investigated in this study. In the reference emmetropic conditions (Fig. 1a), the vitreous chamber is simulated as a sphere (24 mm in diameter), with a planar cap on the anterior side mimicking the indentation of the lens. The geometry in the presence of a posterior staphyloma was obtained by adding posteriorly a spherical volume as shown in Figure 1b. Figure 1c shows the morphology simulating the application of an encircling scleral band on the eye with staphyloma. For each of the three cases, three positions of the patient were analyzed: standing, 45° tilt upward, and supine.

SiO (1000-mPa·s dynamic viscosity) was utilized as a tamponade. Because the SiO used here is composed of 100 % of polydimethylsiloxane, it has a negligible elastic component and a nearly constant shear viscosity^7^ (rheological measurements^8^ show variation in shear viscosity as low as ±4.5% around the average value in the range of 5–50 s^−1^), and thus is assumed to behave as a Newtonian fluid. Hence, our computational model adopted Newtonian constitutive laws for both SiO and aqueous.^3^ Different volumetric ratios of tamponade and aqueous were considered in the vitreous chamber. Specifically, SiO fills from 80% to 100% at 5% increments were considered.

The multiphase flow was simulated, under the hypothesis of incompressible, isothermal, and immiscible fluids, by means of the volume of fluid technique (VOF), which is based on coupling the continuity and momentum equation with an additional conservation equation for the fluid mixture.^9^ More details on the computational method are provided in the Appendix. No-slip was imposed at the walls as a boundary condition for velocity, whereas a dynamic contact angle model was assumed to mimic the behavior of the water–oil–retina interface at the retinal surface. These boundary conditions are physically consistent and accurate in the possible presence of a few-microns-thick layer of fluid in between the tamponade and the retinal surface, the effect of which, together with the wall roughness, should be included among the uncertainties of the model. The static contact angle was derived from the literature,^10^ and the receding and advancing angles were inferred from the experimental work by Chan et al.^11^ Their values are listed in Table 1.

**Table 1.** Physical Properties of Involved Fluids

	SiO	Aqueous
Density	980 kg/m^3^	997 kg/m^3^
Dynamic viscosity	1000 mPa·s	1 mPa·s
Interfacial tension vs. water	0.044 N/m	—
Static contact angle vs. water and retina	16.2°	—
Dynamic contact angle (advancing)	21.2°	—
Dynamic contact angle (receding)	11.2°	—

For each simulation, the tamponade interface was initially set horizontal, and the model was then run until static equilibrium was reached. In that condition, the SiO–aqueous contact surface areas were measured. Afterward, the eye rotated following the same polynomial law as that reported in Rossi et al.^3^:

$$\begin{array}{c} \theta \left(t\right)=c_{0}+c_{1}t+c_{2}t^{2}+c_{3}t^{3}+c_{4}t^{4}+c_{5}t^{5} \end{array}$$

where *t* is time, θ is angular displacement, and the coefficients *c*_1_,…,*c*_4_ were chosen to reproduce a realistic saccade (reported in Table 2).^3^ The saccade lasted 0.137 second, but the simulation was run for an additional 0.137 second to observe the fluid deceleration following the saccadic motion.

**Table 2.** Saccadic Wave Polynomial Coefficients

*c*_0_ (Deg)	*c*_1_ (Deg/s)	*c*_2_ (Deg/s^2^)	*c*_3_ (Deg/s^3^)	*c*_4_ (Deg/s^4^)
0	0	2.01 × 10^4^	−3.29 × 10^5^	2.30 × 10^6^

### Retinal Surface Segmentation

Figures 1d to 1i show the eye regions considered for the analysis:

• Pars plana (green)
• Pre-equatorial retina, the retinal surface anterior to the geometric equator (red)
• Post-equatorial retina, the retinal surface posterior to the geometric equator and between the equator and the macula (blue)
• Macula, the retinal surface centered on the geometric posterior pole and extending for an angle of 20° anteriorly (pale brown)

Each of above regions was further divided into superior and inferior halves.

### Main Outcome Measures

The main study outcome measures include retinal contact measured as the percentage value of retinal surface wet by SiO and the shear stress at the retinal surface.

### Statistical Analysis

ANOVA was applied to statistically evaluate the significance of shear stress values at different locations, as well as to compare the results obtained for the various regions. *P* < 0.05 was considered statistically significant.

## Results

### Overall SiO–Retina Contact

**Figure 2.**
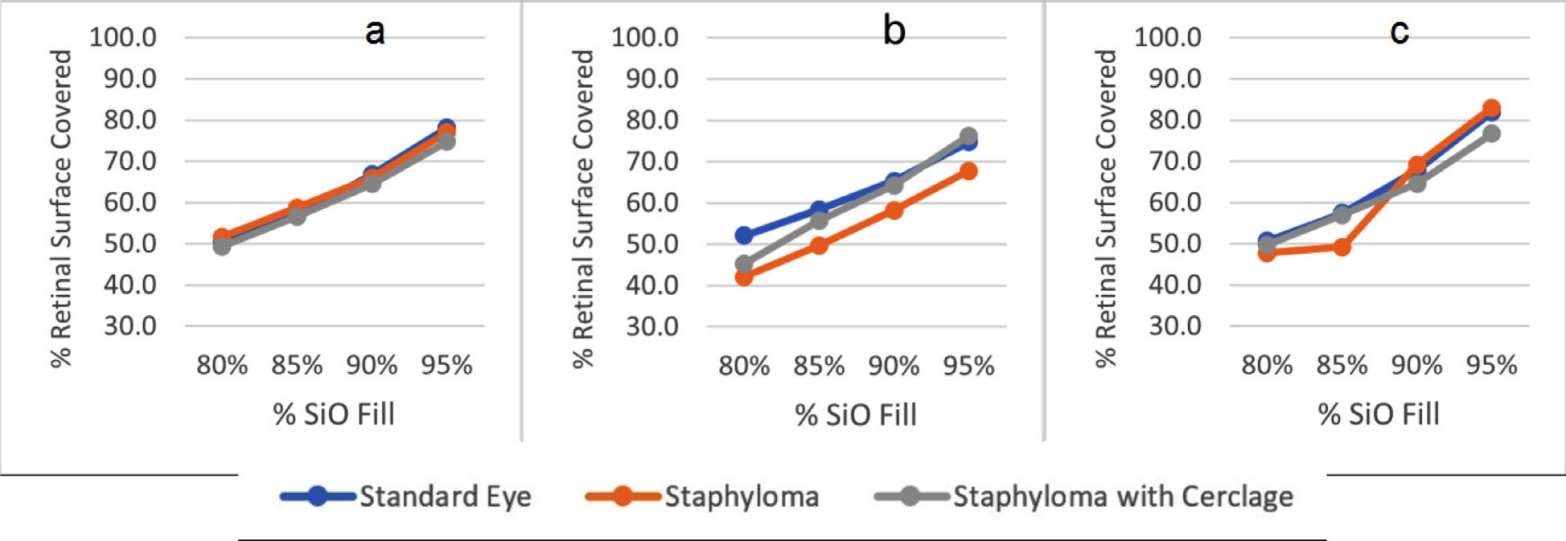
Percentage of retinal surface in contact with the SiO tamponade as a function of the SiO fill percentage for the different geometries. (a) Standing patients, (b) 45° tilt upward patients, and (c) supine patients.

SiO–retina contact is reported as a function of patient position and SiO fill percentage in Figure 2. The presence of an encircling scleral band does not significantly affect the amount of surface contact when the patient is standing (Fig. 2a) or supine (Fig. 2c) and appears mildly disadvantageous irrespective of the fill percentage when the patient is gazing 45° upward (Fig. 2b). Indeed, none of the investigated geometric alternatives significantly affected the overall retinal contact area.

### SiO–Retina Contact by Sectors As a Function of Tamponade Fill

**Figure 3.**
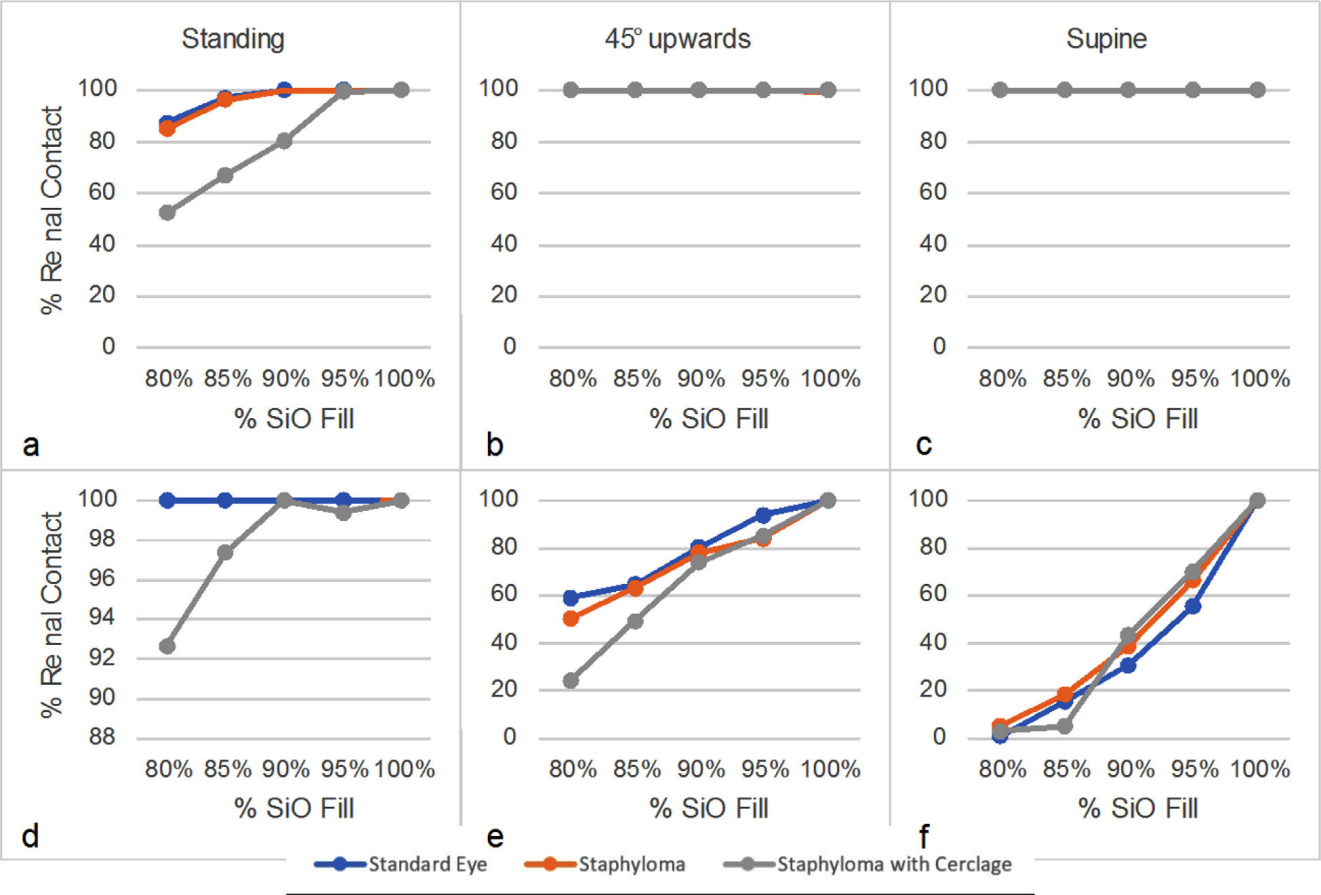
SiO–retina contact of the superior quadrants as a function of fill percentage. (a–c) Pre-equatorial regions for standing patients (a), 45° tilt upward patients (b), and supine patients (c). (d–f) Post-equatorial regions for standing patients (d), 45° tilt upward patients (e), and supine patients (f).

**Figure 4.**
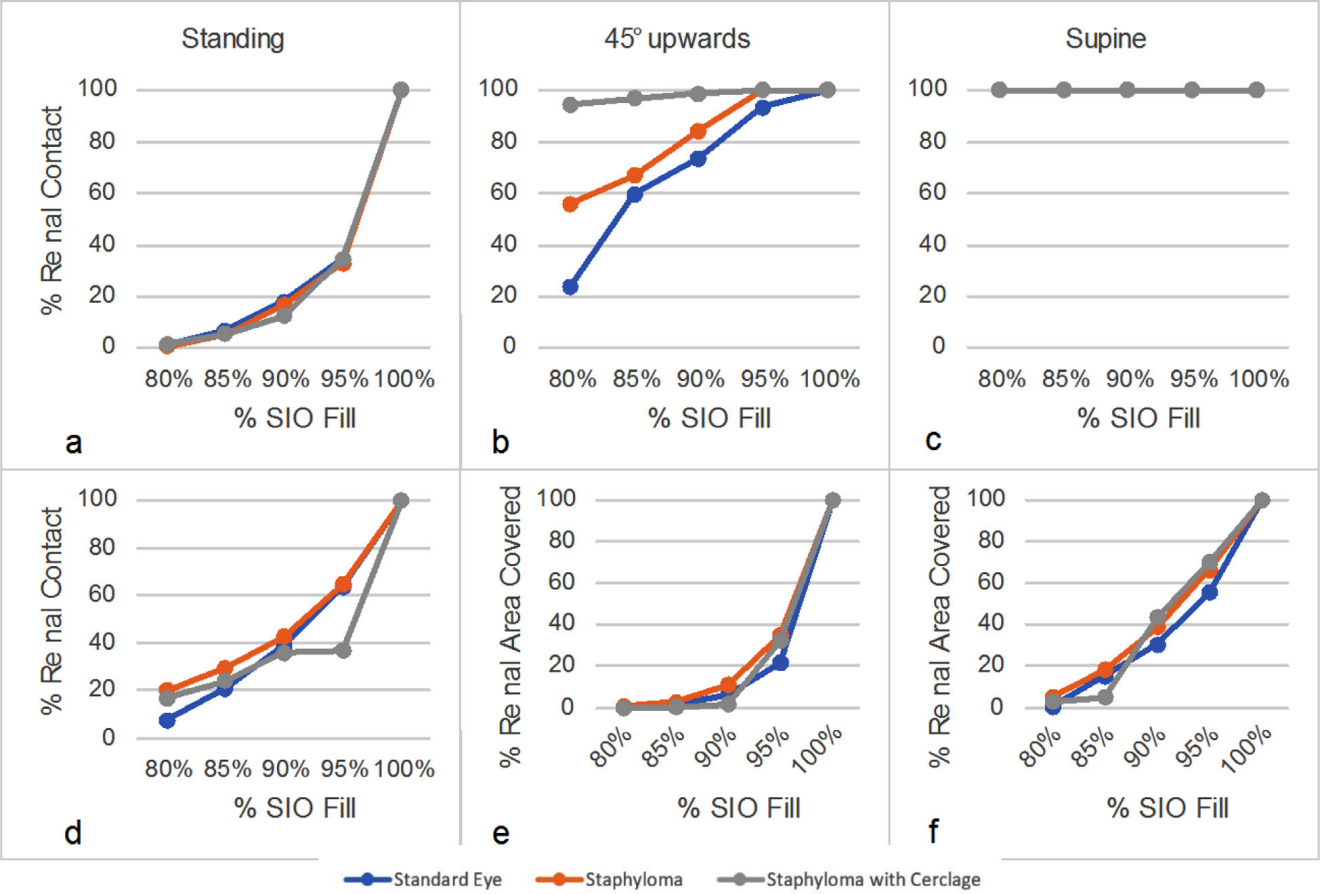
SiO–retina contact of the inferior quadrants as a function of fill percentage. (a–c) Pre-equatorial region for standing patients (a), 45° tilt upward patients (b), and supine patients (c). (d–f) Post-equatorial regions for standing patients (d), 45° tilt upward patients (e), and supine patients (f).

The superior pre-equatorial retina (Figs. 3a–3c) showed extensive SiO contact, especially for fill greater than 85%, and the presence of a scleral band significantly reduced contact only when the patient was standing. The contact of SiO with the superior post-equatorial retina (Figs. 3d–3f) was also reduced in the presence of a scleral band except when the patient was supine, in which case contact was very limited in any case. The percentage of the entire inferior retina in contact with SiO remained well below 50% up to 95% of fill for a standing patient and regardless of vitreous chamber geometry (Figs. 4a, 4d), whereas the presence of an encircling band significantly increased the contact area when the patient gazed 45° upward (Fig. 4b). The entire pre-equatorial retina contacted SiO only when the patient was supine (Fig. 4c), but the inferior post-equatorial retina hardly contacted SiO in any position considered and regardless of variations in geometry (Figs. 4d–4f).

**Figure 5.**
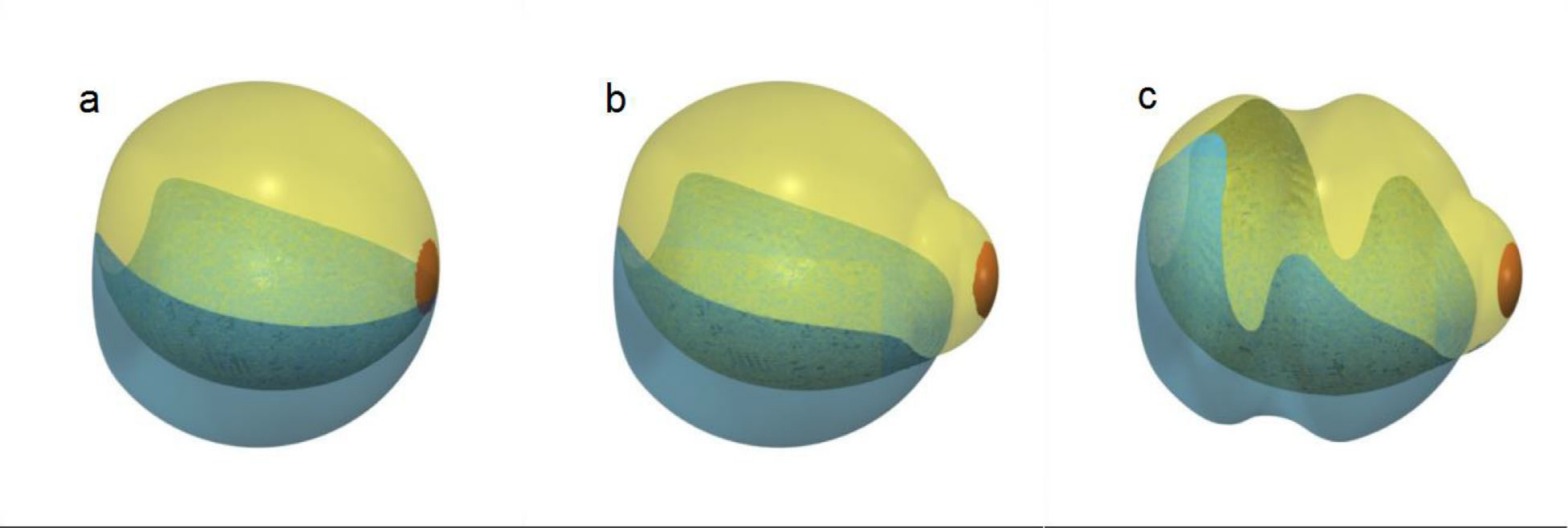
A three-dimensional representation of the SiO interface for a standing patient and an 80% fill percentage. The macula is indicated by the *red area*. (a) Emmetropic eye. (b) Staphyloma. (c) Staphyloma with cerclage.

The presence of an encircling band proved disadvantageous for a standing patient with 95% fill (Fig. 4d). Figure 5 shows that, in the case of a standing patient and emmetropic eye, the macula was only partially wetted by SiO up to an 80% fill. In fact, for a standing patient, the macula remained completely in touch with SiO, irrespective of geometry, only over 85% fill percentage. Conversely, all other tested positions left the macula untouched by SiO up to a 95% filling percentage and unaffected by the presence of an encircling band and/or staphyloma (plots not shown for the sake of brevity).

### SiO–Retina Contact at 90% Tamponade Fill

**Figure 6.**
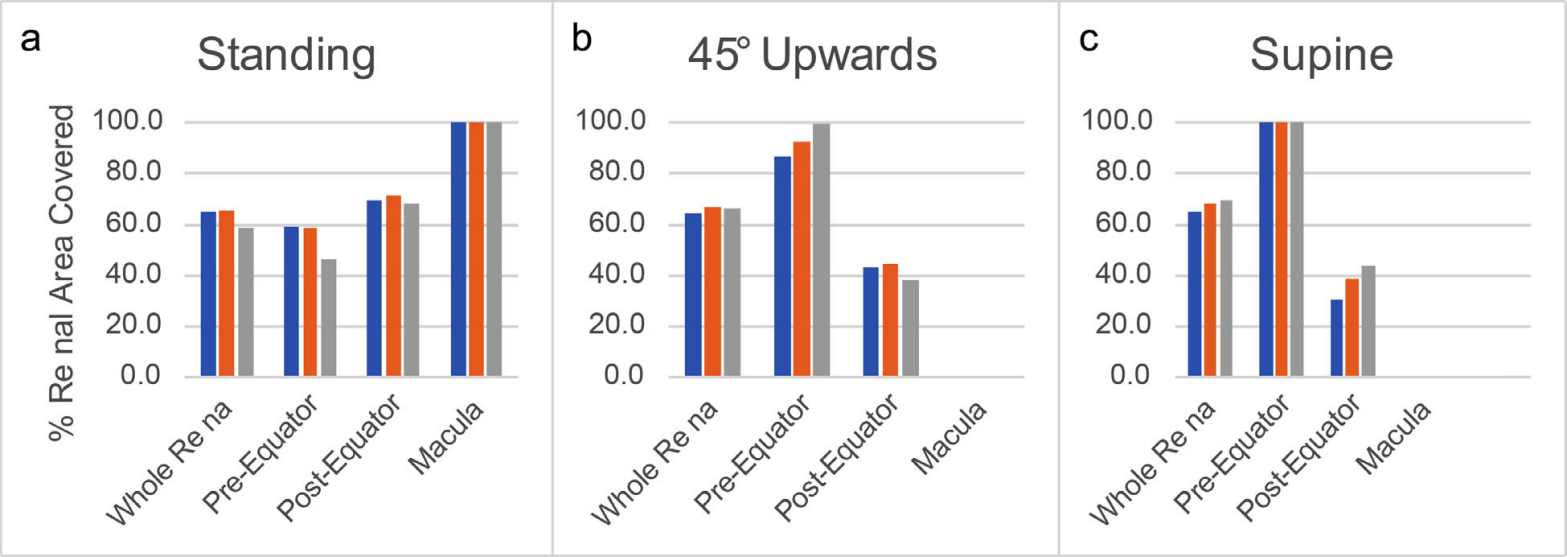
Percentage of regional surface in contact with the SiO at 90% fill percentage: (a) standing patient; (b) 45° upward tilt patient; and (c) supine patient.

A 90% SiO fill was considered representative of the “real-world” post-operative condition; interestingly, the presence of an encircling band reduced the fraction of total SiO–retina contact for a standing patient (Fig. 6a), but it increased contact with the pre-equatorial retina for an upward-gazing patient (Fig. 6b) and with the post-equatorial retina when the patient was supine (Fig. 6c).

Overall, the inferior quadrants showed limited SiO contact for a standing patient, even with a 90% tamponade fill (Fig. 3), and the presence of an encircling band reduced it further. Upward-gaze and supine positioning uncovered the posterior quadrants, as expected (Figs. 3, 6), whereas the encircling band offered little if any advantage for the pre-equatorial retina in the upward gaze.

The macula completely contacted SiO only for a standing patient and was entirely wetted by aqueous at the 45° upward tilt and supine positions, regardless of the presence of an encircling band. Supine position almost completely prevented SiO from getting into touch with the post-equatorial retina, including the macula (Figs. 3^4^,^5^–6).

### Wall Stress During the Saccade

**Figure 7.**
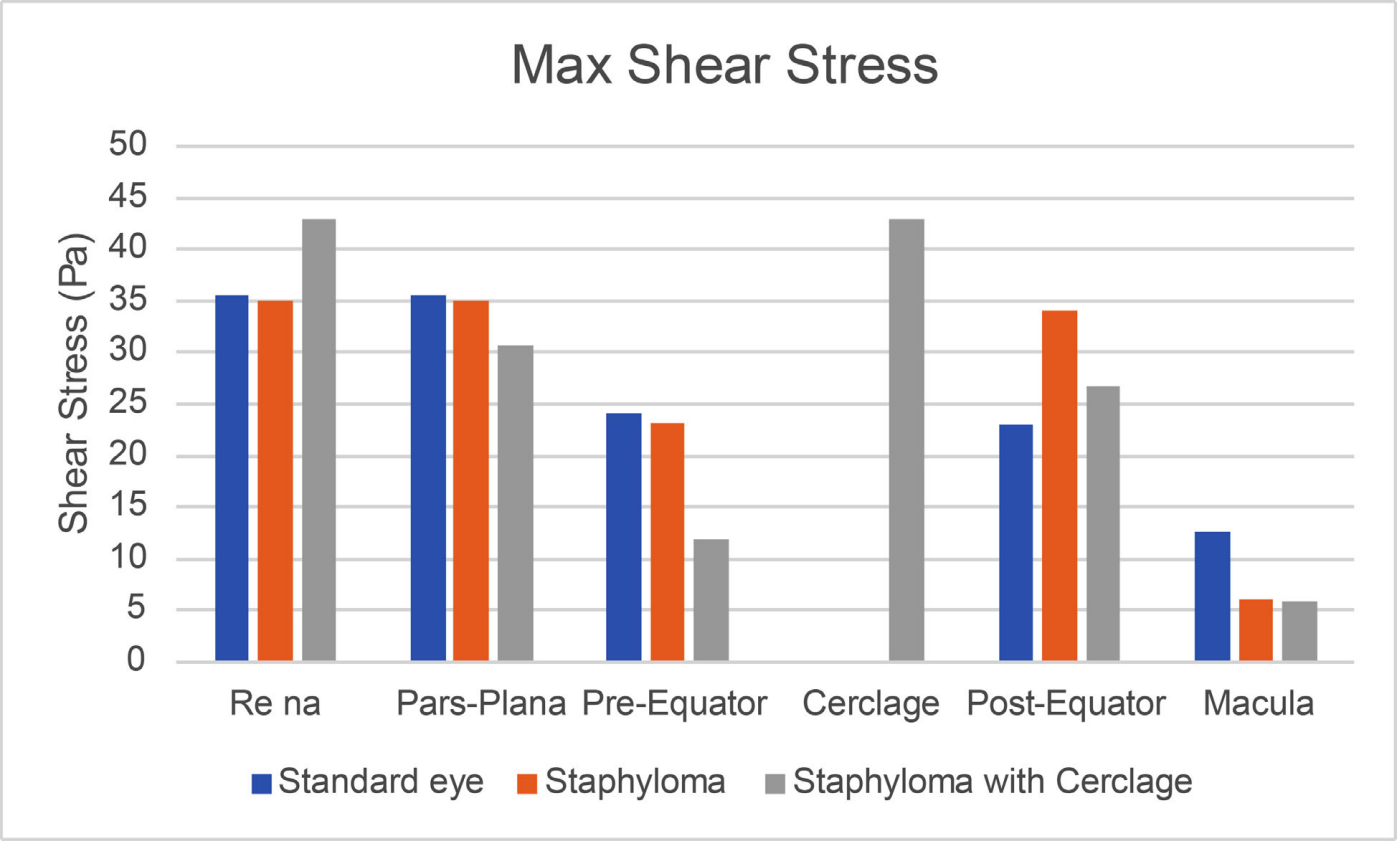
Maximum shear stress in the various regions for a standing patient and the Silicone Oil 1000 centistokes (SiO1000).

**Figure 8.**
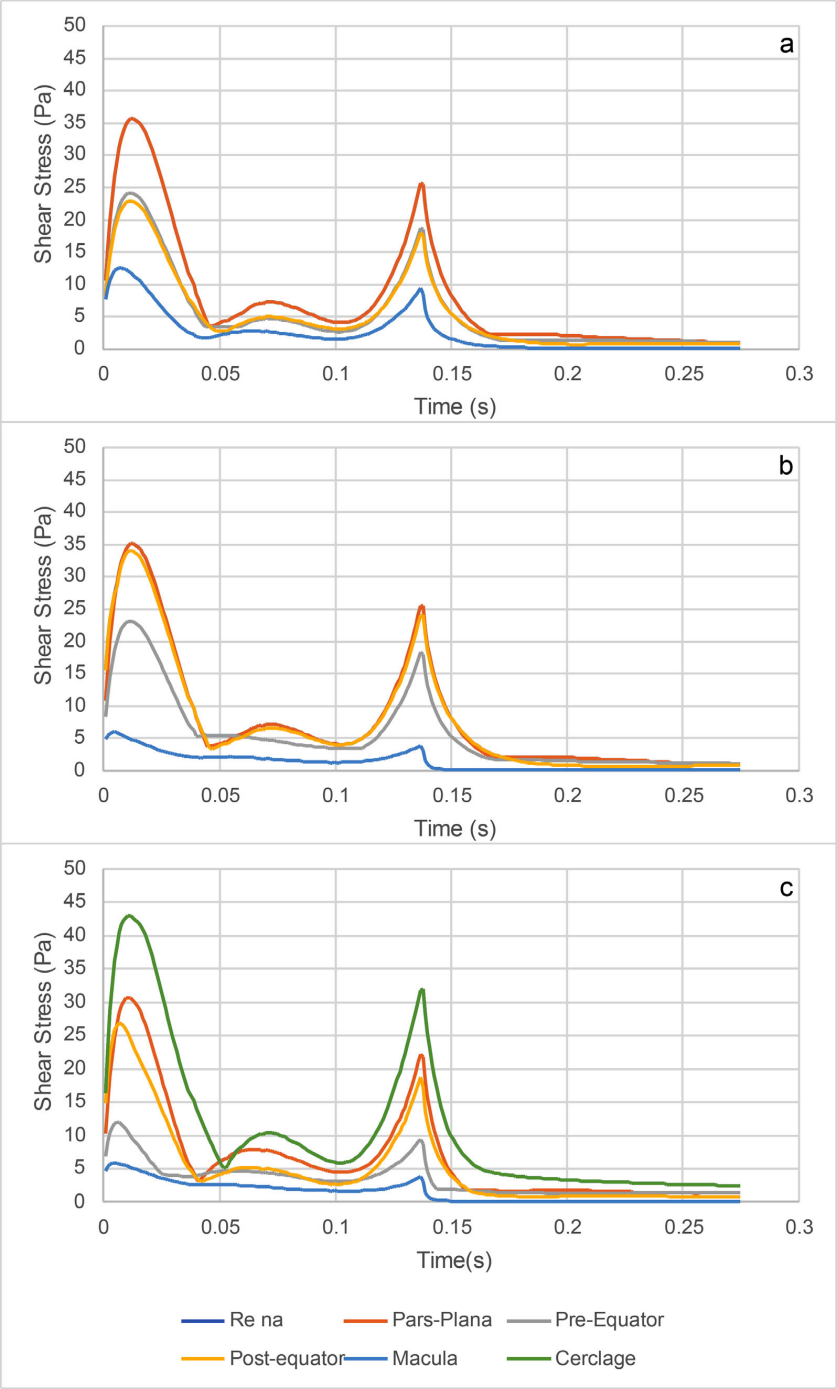
Instantaneous maximum shear stress (IMSS) for the SiO1000 and a standing patient in the different regions of the eye: (a) emmetropic eye; (b) staphyloma; and (c) staphyloma with an encircling scleral band.

In order to characterize the tangential forces exerted by the SiO on the retina during the saccade, we considered the case of a standing patient with a 90% fill and compared the three geometries, analyzing the values of shear stress reached during the simulation time. The maximum shear stress attained in each region during the entire observation time is plotted in Figure 7. The corresponding pointwise values are represented as three-dimensional maps in Supplemental Material SDC1. The distribution of the maximum pressures attained during the saccade in the case of 90% fill are displayed in Supplemental Material SDC2 for the three geometric variants. Pressure values seem to be driven mainly by inertial effects: the highest pressure magnitudes are located around the horizontal midplane and, in particular, in the indentations generated by the staphyloma and the cerclage, whereas the pressure values are lower in the region wet by the water. The time evolution of the maximum tangential forces is plotted in Figure 8, which shows the value of the maximum shear stress attained over each eye region at each time instant. Analysis of Figure 7 shows that, in the presence of the staphyloma, the post-equatorial retina was exposed to a significantly higher maximum shear stress as opposed to the macula, where shear stress reached a minimum irrespective of the geometry. The presence of an encircling band shifted most of the high shear stresses from the pre-equatorial retina to the area of the indentation generated by the encircling band, where the shear stress attained its absolute maximum.

**Figure 9.**
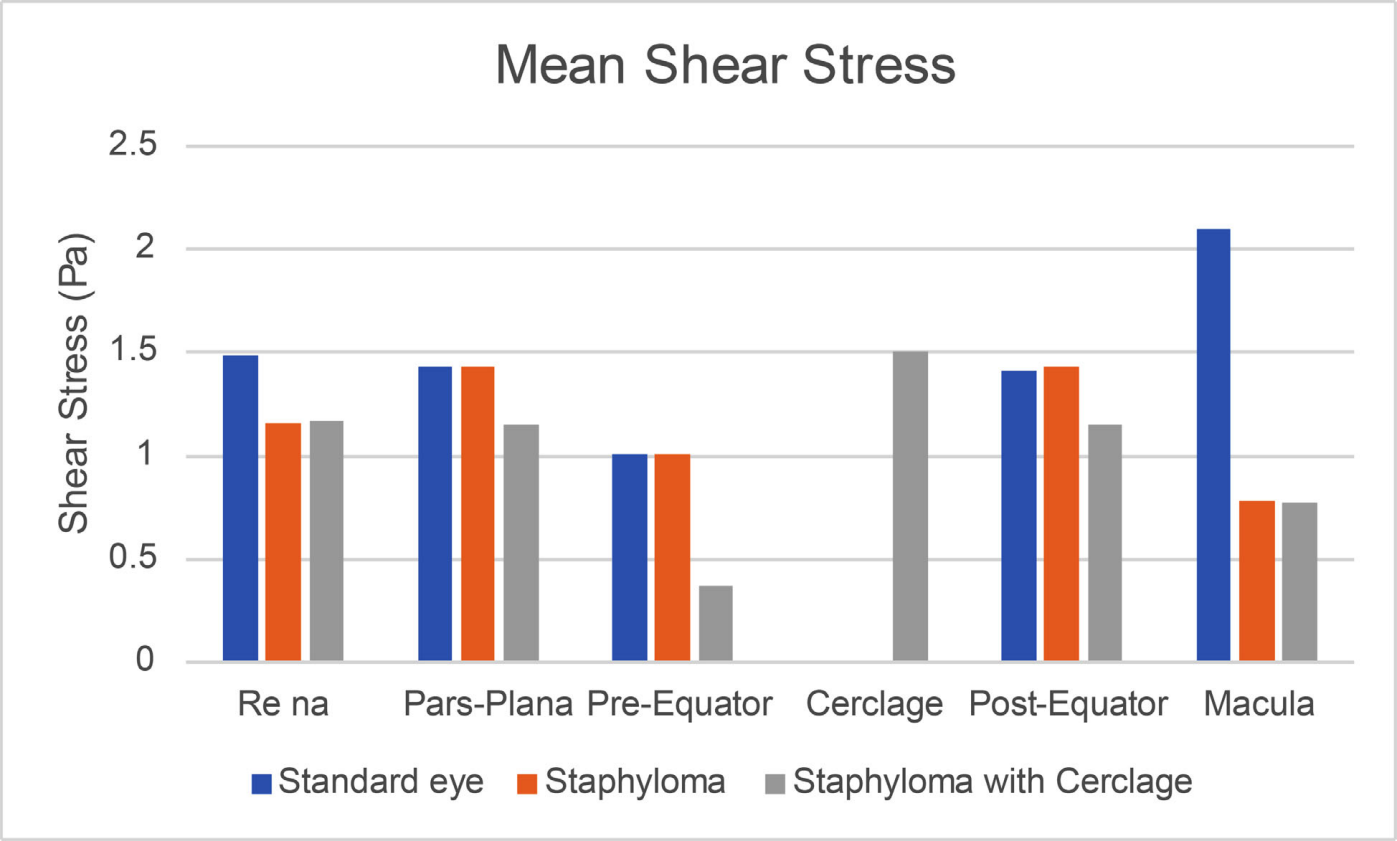
Shear stress averaged over each region and the simulation time.

**Figure 10.**
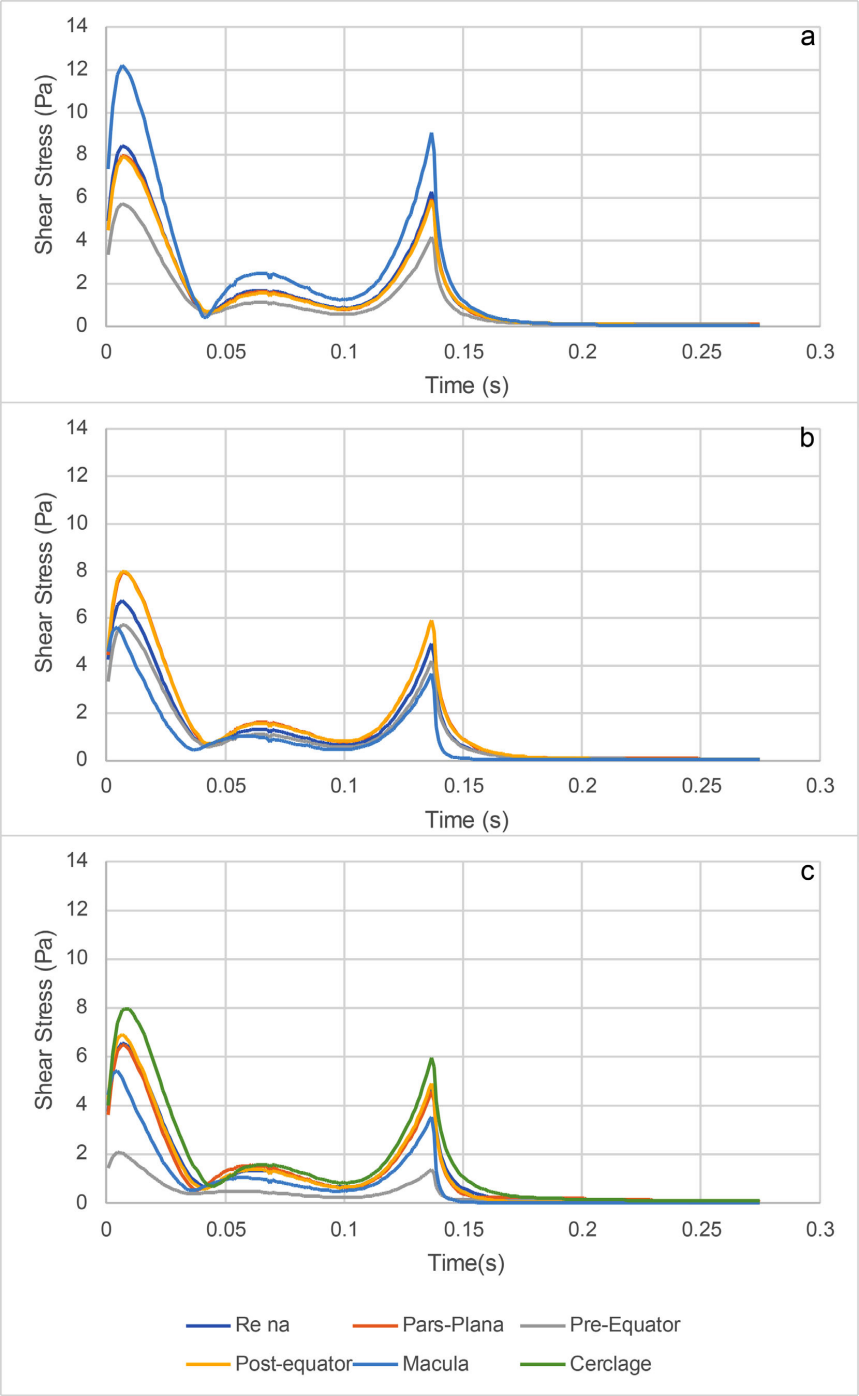
Instantaneous average shear stress (IASS) for the SiO1000 and a standing patient in the different regions of the eye: (a) emmetropic eye; (b) staphyloma; and (c) staphyloma with a 360° scleral band.

The average shear stress over each region and during the entire observation time, displayed in Figure 9, was reduced significantly in the macula in the presence of a staphyloma compared to the emmetropic eye, in agreement with the behavior of the instantaneous values of the shear stress, averaged over each region, reported in Figure 10. The addition of an encircling scleral band increased the average shear stress at the scleral indentation and significantly reduced the stress on the pre-equatorial retina.

## Discussion

Reaching and maintaining the broadest possible contact between the retina and SiO have been regarded as being pivotal in reducing the risk of retinal re-detachment. SiO effectively keeps aqueous away from retinal tears, preventing its leakage into the subretinal space, and reduces the volume of aqueous reservoirs where inflammatory mediators pool, thus decreasing the risk of proliferative vitreoretinopathy (PVR).^12^

The present paper describes SiO statics and dynamics and its interaction with the retina under the most frequent pathologic or iatrogenic conditions altering the vitreous chamber contour (the presence of a posterior staphyloma with the possible application of a 360° scleral indentation), compared to an idealized emmetropic eye. In our CFD model, SiO fill percentage proved to be the most important single factor impacting the extension of the retinal contact. Its increase from 80% to 95% in volume significantly increased the overall retinal contact area regardless of patient positioning and ocular geometry variations (Fig. 2).

Interestingly, an 80% SiO fill contacted only a little more than 50% of the entire retinal surface regardless of the eye geometry and positioning, and even a 95% volume fill left about 20% of the retinal surface untouched (Fig. 2). It should be noted that myopic eyes have a greater retinal surface than emmetropic eyes (the longer the axial length, the greater the area, being a function of squared radius); thus, the same percentage of retinal surface untouched by SiO in myopic or emmetropic eyes corresponds to a much greater extent in the first case, with higher risk of retinal re-detachment.^13^

The apposition of a scleral band in association with pars plana vitrectomy has been regarded as offering several benefits. It would improve the contact between SiO bubble and the retina,^14^ contrast retinal shortening due to PVR, and re-direct the vector force toward the retinal pigment epithelium to strengthen adhesion.^15^ Romano and colleagues^16^ showed that an encircling band associated with gas tamponade achieved the same success rate as the heavier-than-water Densiron 68 tamponade for inferior retinal detachment, although a surgical series has inconsistently associated scleral indentation with a better anatomical outcome.^17^

Our model indeed shows that the presence of an encircling band does not increase the amount of retina in touch with the tamponade (Fig. 2). Therefore, geometric changes more than contact variation may influence clinical success rate, as scleral indentation undoubtedly contrasts with retinal shortening^18^ and, indirectly, PVR development.^19^^,^^20^ Head positioning is also considered, with the aim of improving SiO–retinal contact, at least in some sectors, but it remains a matter of debate because of the intrinsic difficulty of maintaining the prescribed posture, the uncertain benefits,^21^ dubious compliance, and possible drawbacks.^22^

When analyzing the retinal sectors separately, the inferior retina showed limited contact with SiO; up to a 95% fill rate and the presence of a scleral band significantly improved only pre-equatorial retinal contact when the patient was gazing upward (Fig. 4b). As expected, the superior retina maintained better contact with SiO than the inferior in any position considered, except when the patient was supine (Figs. 3, 4). The presence of a scleral band proved to be disadvantageous, as it significantly reduced the contact area, especially across the post-equatorial region when the patient was supine or gazing upward, whereas the staphyloma did not influence the contact area at all.

Overall, our model does not support the hypothesis that 360° scleral indentation improves SiO–retinal contact, except for a few notable exceptions regarding specific sectors and eye positions of uncertain clinical relevance. More importantly, even when a 90% SiO fill was considered, none of the retinal sectors maintained a satisfying tamponade contact, regardless of the positioning.

Myopic staphyloma did not impact SiO–retinal contact significantly in any of the cases considered. The macula maintained good contact with SiO only for a standing patient, whereas all of the other positions left it completely wetted by aqueous even with 90% SiO fill; the staphyloma and scleral band made no difference in any such cases (Fig. 6). Shear stress distribution during a saccade greatly varied throughout the considered sectors as ocular geometry changed. The staphyloma reduced maximum and mean shear stress at the macula compared to emmetropic geometry, whereas in the presence of a scleral band shear stress increased over the indentation and was reduced elsewhere (Fig. 7). The shear stress variation remains of uncertain clinical relevance, although it is conceivable that higher shear stress may favor tangential displacement of the retina and destabilize its adhesion to the retinal pigment epithelium, as retinal shifting after vitrectomy has been described previously.^23^

In summary, our model shows that the highest possible SiO fill is mandatory, as even 90% fill leaves almost 30% of the retinal surface untouched, regardless of a patient's position. Moreover, the current outcomes suggest that the introduction of new tamponades contacting the entire retina would be highly desirable.^24^ A 360° scleral band does not increase SiO contact, although it may contrast retinal shortening and traction vectors, and it certainly promotes a significant shear stress increase at the indentation site. The pitfalls of the present study are primarily related to the uncertainties of SiO–retina static and dynamic interactions, as well as the deviation of normal human anatomy from the standardized vitreous chamber shape adopted here.

## Supplementary Material

Supplement 1

Supplement 2

## Appendix: Computational Methods

In this study, the unsteady two-phase flow of tamponade and aqueous is modeled by means of the public-domain CFD code OpenFOAM, under the hypothesis of incompressible, isothermal, immiscible fluids. The governing equations of fluid mechanics are discretized using the finite volume approach and solved simultaneously for aqueous and silicone by means of the volume of fluid (VOF) method, which is based on introducing the volume fraction α, which is the ratio of the volume occupied in a cell by one fluid (in our case the water) to the cell volume. In the VOF method, the density ρ is defined as

$$\begin{array}{c} \rho =\alpha \rho_{1}+\left(1-\alpha \right)\rho_{2} \end{array}$$

where the volume fraction α ranges from 1 inside fluid 1 (the aqueous) having density ρ_1_ to 0 inside fluid 2 (the tamponade) having density ρ_2_. The governing equations read as follows:

$$\begin{array}{c} \frac{\partial u_{j}}{\partial x_{j}}=0 \end{array}$$

$$\begin{array}{c} \frac{\partial }{\partial t}\left(\rho u_{i}\right)+\frac{\partial }{\partial x_{j}}\left(\rho u_{i}u_{j}\right)=-\frac{\partial p}{\partial x_{i}}+\rho f_{i}+\mu \frac{\partial u_{i}}{\partial {x_{j}}^{2}}+\rho f_{\sigma i} \end{array}$$

The former states the conservation of mass (the so-called continuity equation), and the latter represents the momentum balance (namely, the Navier–Stokes equation). In these equations, µ is the dynamical viscosity, *p* is the pressure, *u*_i_ is the *i*th velocity component, and *f_i_* and *f*_σ_*_i_* are the gravitational and the surface tension forces per unit mass, respectively. Continuity and Navier–Stokes equations are coupled with a transport equation for the aqueous volume fraction:

$$\begin{array}{c} \frac{\partial \alpha }{\partial t}+\frac{\partial }{\partial x_{j}}\left(\alpha u_{j}\right)=0 \end{array}$$

The above equations were numerically solved on the computational grid using spatially second-order accurate schemes, whereas temporal advancement of the flow and of the volume fraction was performed using the implicit, first-order accurate Euler scheme. The solution of the pressure–velocity coupling was obtained through the pressure-implicit with splitting of operators (PISO) method.^25^

The fluid domain was discretized with an unstructured mesh consisting of hexahedral and tetrahedral cells, using the grid generation tool snappyHexMesh, included in the OpenFOAM library. Following an independence grid sensitivity test, the adopted meshes had around 350,000 cells, and they were characterized by five layers of extruded hexahedral cells at the eye surface, with a maximum stretching ratio of 1.1. The dynamic mesh technique was adopted, and the grid was rigidly rotated according to the law of motion reproducing the saccade.

## Supplementary Material

SDC 1. Pointwise maximum shear stress values over each region shown as 3D map.

SDC 2. Distribution of the maximum pressures attained during the saccade for the three geometries and 90% fill.
